# Malignant phyllodes tumor arising from a previously biopsy-proven fibroadenoma: a case report

**DOI:** 10.1093/jscr/rjag478

**Published:** 2026-07-22

**Authors:** Archana Venkatesan, Anna Brown, Navya Katragadda, Katheryn Light, Catherine Ronaghan

**Affiliations:** Medical College of Georgia, 1120 15th St, Augusta, GA 30912, United States; Medical College of Georgia, 1120 15th St, Augusta, GA 30912, United States; Medical College of Georgia, 1120 15th St, Augusta, GA 30912, United States; Medical College of Georgia, 1120 15th St, Augusta, GA 30912, United States; Medical College of Georgia, 1120 15th St, Augusta, GA 30912, United States

**Keywords:** malignant phyllodes tumor, fibroadenoma, fibroepithelial lesion, malignant transformation, breast neoplasm, core-needle biopsy

## Abstract

Fibroadenomas are common benign lesions that are typically managed conservatively. However, transformation into malignant phyllodes tumor is a rare but critical progression that demands a shift from surveillance to surgical intervention. We present the case of a patient who underwent multiple core-needle biopsies over several years, all confirming a fibroadenoma, yet the mass demonstrated progressive growth, ultimately exceeding 7 cm. Ultimately, surgical excision revealed a malignant phyllodes tumor characterized by stromal overgrowth, cellular atypia, increased mitotic activity, and infiltrative margins. This case highlights the diagnostic limitations of core-needle biopsy in fibroepithelial lesions. Although the vast majority of fibroadenomas remain benign, a minority may undergo malignant transformation. Physicians must maintain a high degree of clinical suspicion and closely monitor longstanding lesions for any signs of growth or change. Early distinction between fibroadenomas and phyllodes tumors is essential, as management strategies differ substantially.

## Introduction

Fibroadenomas are benign breast pathologies found in women, typically described as painless, mobile, unilateral nodules of fibrous tissue with regular borders [1]. Diagnosis involves physical examination, ultrasonography or mammography, and additional confirmatory core-needle or fine-needle biopsy [2]. Fibroadenomas are typically managed conservatively, with current guidelines suggesting a 1 to 3-year follow-up for non-palpable lesions. Palpable lesions are monitored with imaging every 6 months until age 35 or excised if symptomatic or poorly tolerated [3].

While malignant transformation is rare, rapid growth may warrant additional testing and more rigorous follow-up [4]. In rare instances, clonal progression may result in the development of either carcinoma *in situ* or phyllodes tumors [5].

Phyllodes tumors are rare fibroepithelial neoplasms with leaf-like epithelial clefts, which may range from benign to malignant. In particular, malignant phyllodes tumors exhibit marked nuclear pleomorphism, increased stromal cellularity and mitotic activity, and infiltrative borders [6]. With a 5-year survival rate of 80% and a high rate of local and distant relapse, they are aggressive tumors, managed with wide local excision to achieve clear surgical margins [7].

This case illustrates a rare but clinically significant instance of malignant transformation from a biopsy-proven fibroadenoma to a malignant phyllodes tumor. It highlights the importance of surveillance and clinical judgment when managing breast masses that deviate from expected behavior.

## Case presentation

The patient is a female in her 7th decade who presented to an outpatient surgical oncology clinic for evaluation of an enlarging mass in the upper outer quadrant of the right breast. The patient reported right axillary tenderness but denied generalized mastalgia, nipple discharge, and/or additional palpable masses or nodules. Physical exam revealed a tender 8 × 8 cm mass and profound bilateral grade 3 ptotic macromastia, with the right breast weighing 100 g more than the left. No skin changes or pathologic lymphadenopathy were found. The left breast revealed no abnormalities other than an inferior periareolar scar.

Her medical history included a left lumpectomy (25 years prior), ovarian cyst removal, and hypertension. She had three pregnancies, two of which resulted in live births, and reached menopause at age 55. The patient used oral contraceptives for 1 year and hormone replacement therapy for 7 years. Family history was notable for unspecified cancer, and she reported occupational exposure to fertilizers and pesticides.

In 2021, the patient underwent a diagnostic digital mammogram that revealed calcifications in the posterior lower inner quadrant of the right breast. Based on these findings, a vacuum-assisted stereotactic-guided biopsy with clip placement was performed. Pathology revealed a fibroadenomatoid nodule with calcifications, benign breast tissue, and a 1.9 cm fibroadenoma.

Three years later, the mass had enlarged to 2.7 cm. Bilateral mammography and targeted ultrasound confirmed a lesion at the 11 o’clock position, 4 cm from the nipple. Core biopsy demonstrated fibroadenoma with BI-RADS 4 classification, and repeat biopsy one month later again confirmed fibroadenoma.

One year later, diagnostic mammography, targeted ultrasound, and breast magnetic resonance imaging demonstrated interval growth to a dominant 6.6 × 5.7 cm circumscribed mass with stable clustered benign calcifications at the 11 o’clock position. Imaging remained BI-RADS 4, and phyllodes tumor was considered. No abnormal axillary or internal mammary lymph nodes were identified.

**Figure 1:**
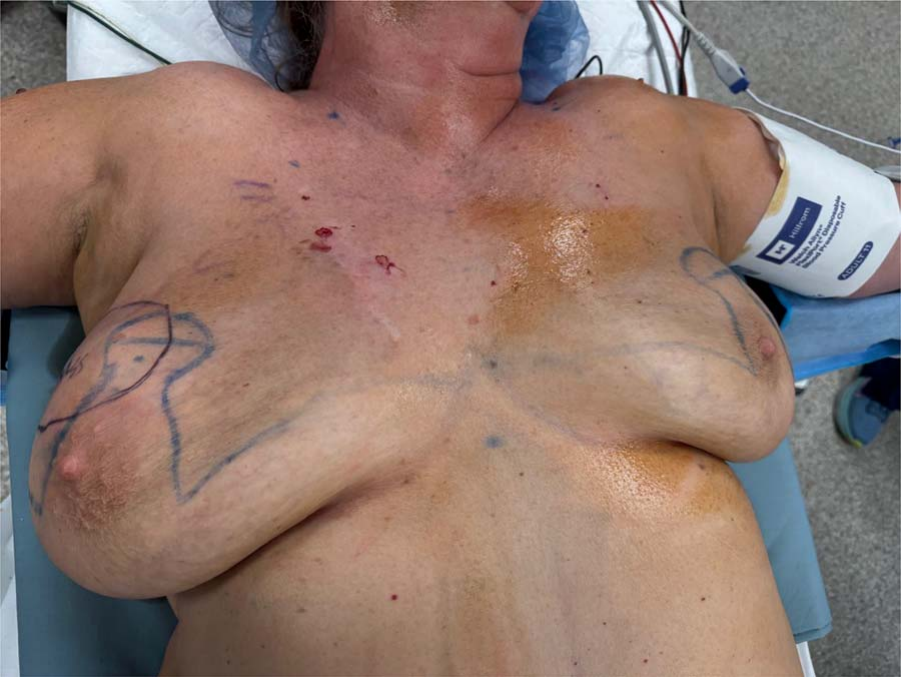
Anterior chest view demonstrating marked right breast enlargement with asymmetric contour and pre-surgical markings outlining planned resection.

**Figure 2:**
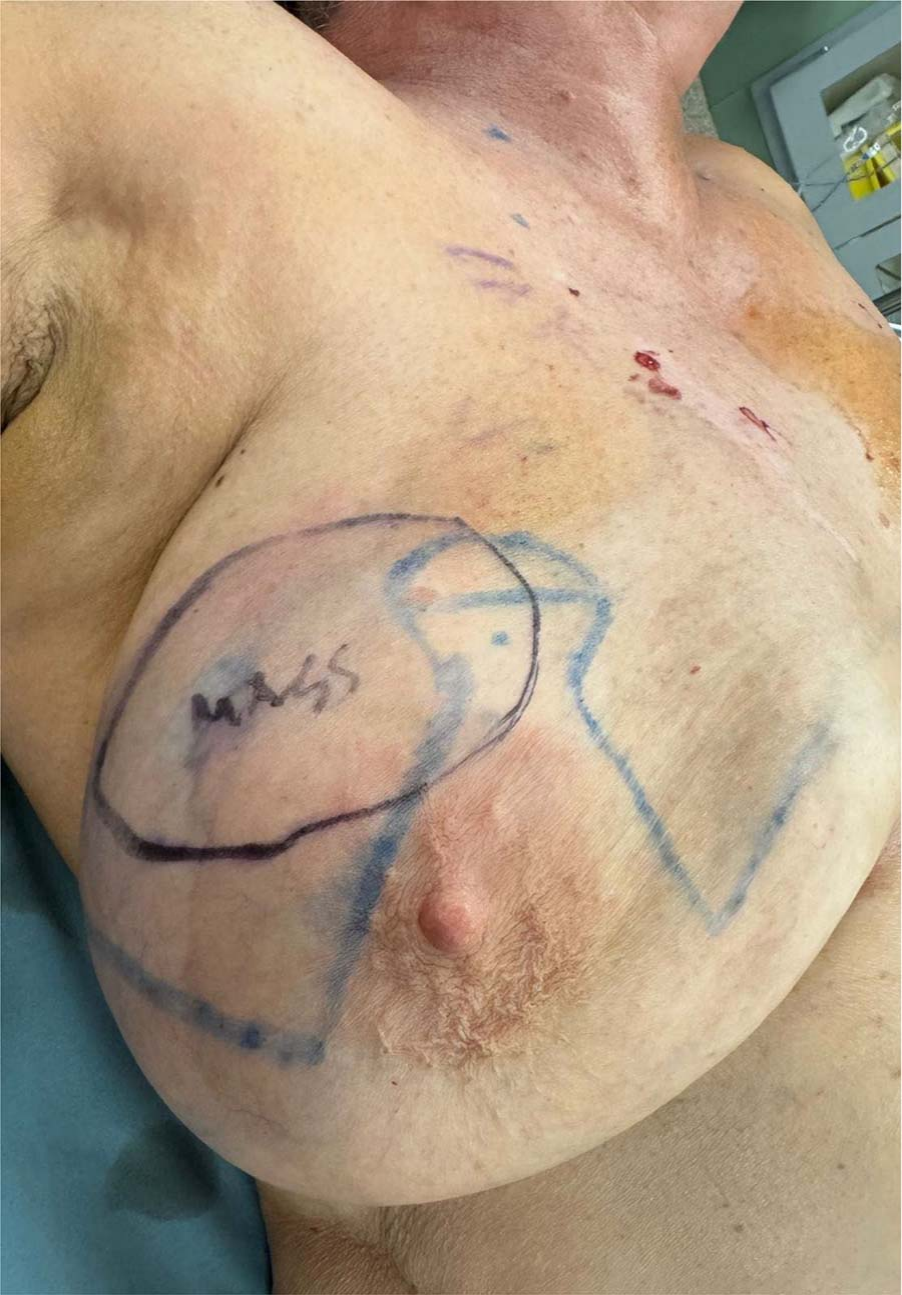
Preoperative close-up of the breast demonstrating significant enlargement and surgical markings outlining the mass and planned incisions.

The patient subsequently underwent a right partial mastectomy with oncoplastic reconstruction, along with a balancing left breast reduction mammoplasty (Figs 1 and 2). Intraoperative optical coherence tomography margin analysis of the right partial mastectomy specimen revealed microcalcifications and annular features at the posterior lateral margins. Lateral and posterior margins were otherwise clear, and no lymph node specimens were provided. Anatomic pathology revealed a well-circumscribed 7.5 × 6 × 6 cm pink-tan fleshy mass with cystic areas containing serosanguineous fluid up to 2.2 cm and focal gelatinous regions. Histopathological evaluation showed a fibroepithelial lesion with stromal overgrowth, moderate stromal atypia, moderate stromal cellularity, and infiltrative histologic tumor border with a mitotic rate of 17 mitoses per 10 HPFs. The final diagnosis was a 7.5 × 6.6 cm malignant phyllodes tumor, staged as pT2 pNX. The patient was discharged and was advised to follow up with surgical oncology.

## Discussion

Differentiating between fibroadenomas and phyllodes tumors is crucial because of their markedly different prognoses and treatment approaches. While fibroadenomas require minimal intervention, phyllodes tumors demand more nuanced management due to their potential for metastasis (seen in ~25% of malignant cases) [7]. Unfortunately, they can be difficult to distinguish due to similar histopathological and sonographic features. In one cohort, 13% of excision-proven phyllodes tumors were initially misdiagnosed as fibroadenomas on core biopsy [8]. Thus, there is clearly a potential for misdiagnosis.

In the present case, however, misdiagnosis is less likely, as repeated biopsies consistently demonstrated findings consistent with fibroadenoma. This leaves the possibility of malignant transformation of the fibroadenoma into a phyllodes tumor. While the exact molecular mechanisms behind this transformation are unknown, both FA and phyllodes tumors frequently exhibit MED12 mutations, suggesting a possible shared origin [9]. Subsequent malignant transformation may be associated with mutations in TERT and TP53.

As such, heightened vigilance through regular follow-up and thorough examination may aid in earlier detection and management. This is especially relevant in older patients, as increasing age is associated with more complex histologic features and malignant potential in fibroepithelial lesions. The median age for simple fibroadenomas is ~28.5 years, compared with 47 years for complex fibroadenomas, reflecting a greater likelihood of atypia, stromal overgrowth, or malignant transformation [10]. Thus, persistent or rapid growth, especially in older patients, should prompt reevaluation for possible complications such as transformation to phyllodes tumor or malignancy.

This case underscores the importance of surveillance in patients with fibroadenomas. While the vast majority remain benign, a minority of cases may undergo malignant transformation. Physicians must maintain a high degree of clinical suspicion and closely monitor longstanding lesions for any signs of growth or change. Molecular profiling, such as evaluation of MED12, TERT, and TP53 status, may aid in risk stratification and help guide treatment protocol as well. Early detection and proactive management are key to preventing malignant transformation and improving survival outcomes.
